# Investigating dynamical deformations of tumor cells in circulation: predictions from a theoretical model

**DOI:** 10.3389/fonc.2012.00111

**Published:** 2012-09-18

**Authors:** Katarzyna A. Rejniak

**Affiliations:** ^1^ Integrated Mathematical Oncology, H. Lee Moffitt Cancer Center and Research InstituteTampa, FL, USA; ^2^ Department of Oncologic Sciences, College of Medicine, University of South FloridaTampa, FL, USA

**Keywords:** circulating tumor cells, metastatic cascade, cell deformation, computational modeling, immersed boundary method

## Abstract

It is inevitable for tumor cells to deal with various mechanical forces in order to move from primary to metastatic sites. In particular, the circulating tumor cells that have detached from the primary tumor and entered into the bloodstream need to survive in a completely new microenvironment. They must withstand hemodynamic forces and overcome the effects of fluid shear before they can leave the vascular system (extravasate) to establish new metastatic foci. One of the hypotheses of the tumor cell extravasation process is based on the so called “adhesion cascade” that was formulated and observed in the context of leukocytes circulating in the vascular system. During this process, the cell needs to switch between various locomotion strategies, from floating with the blood stream, to rolling on the endothelial wall, to tumor cell arrest and crawling, and finally tumor cell transmigration through the endothelial layer. The goal of this project is to use computational mechanical modeling to investigate the fundamental biophysical parameters of tumor cells in circulation. As a first step to build a robust *in silico* model, we consider a single cell exposed to the blood flow. We examine parameters related to structure of the actin network, cell nucleus and adhesion links between the tumor and endothelial cells that allow for successful transition between different transport modes of the adhesion cascade.

## INTRODUCTION

The metastatic cascade is a multistep process in which cancer cells from the primary side (of the solid tumor) are relocated to the distant organs in which they grow to form new colonies. This process, called a metastatic cascade, consists of several steps during which cancer cells must first invade the stroma surrounding the primary tumor, intravasate, i.e., enter into the blood or lymphatic system, survive in the circulation, extravasate into the secondary site, invade the local matrix, and grow in the target organ (Chambers et al., 2002; Nguyen et al., 2009; Psaila and Lyden, 2009; Mina and Sledge, 2011).

The tumor cells in circulation (called the circulating tumor cells, CTCs) are exposed to various microenvironmental factors that are novel for the cells arising from the solid tumor mass. In order to survive, the cells must withstand hemodynamic forces and overcome the effects of fluid shear (Wirtz et al., 2011). In order to metastasize, the cells must leave the circulation system, and one of the extravasation hypotheses is the so called “adhesion cascade” that was formulated and observed in the context of circulating leukocytes (reviewed in Nourshargh et al., 2010). During this process, the tumor cell needs to switch between various locomotion strategies, from floating with the blood stream, to rolling on the endothelial wall (EW), to tumor cell arrest and crawling, and finally tumor cell transmigration through the endothelial layer (Nourshargh et al., 2010; Wirtz et al., 2011).

The recent advent of technical devices capturing the CTCs from patient’s peripheral blood (Hsieh et al., 2006; Alunni-Fabbroni and Sandri, 2010; Farace et al., 2011; Kirby et al., 2012), allows the cancer biology community to gain enormous knowledge about the biology of CTCs and their correlations with clinical outcomes (Hou et al., 2010; Oakman et al., 2010; Swaby and Cristofanillli, 2011; Wendel et al., 2012). However, many aspects of CTC transport and extravasation processes are still unknown. In particular, the biophysical properties of CTCs that enable the completion of the adhesive cascade have not been investigated in detail, and many questions are still unanswered. How big are the CTCs? How deformable are they? Are the CTC’s biophysical properties similar or distinct from the properties of the primary tumor cells and other cells in the circulation? Do these physical properties change over time? This provides a challenge from both biomechanical and therapeutical perspectives.

The goal of this project is to use mathematical modeling to investigate how cell deformability affects its translocation within the vascular duct under the blood flow, and how the physical properties of the cells change (globally or locally) when the cells switch their locomotion strategies. This will allow us to identify combinations of parameters that can be manipulated experimentally in order to disrupt some steps of the adhesive cascade.

## MATERIALS AND METHODS

**FIGURE 1:**
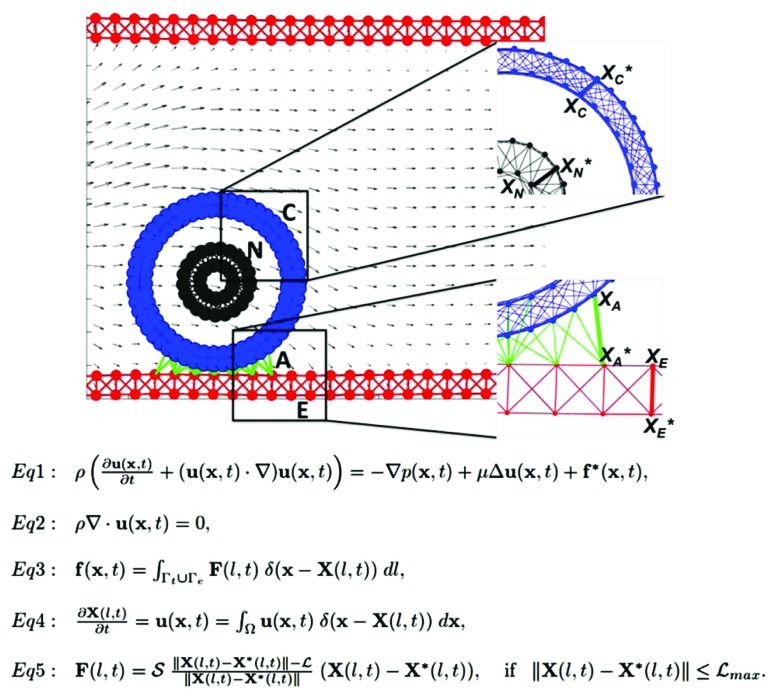
**Model schematics and equations.** A schematic representation of the 2D model of the tumor cell in circulation. The blood vessel (red walls, E) is interpenetrated by a laminar flow (gray arrows representing blood velocity field) that interacts with a circulating tumor cell (CTC). The cell nucleus (gray, N) is surrounded by the cell cortex (blue, C) that both define cell shape and stiffness. The cell near the endothelial wall (EW) develops CTC–EW adhesive connections (green links, A). Insets show the magnification of a meshwork defining the nucleus and the cortex (top), and the network defining the EW and CTC–EW adhesive springs (bottom). Representative springs of the cortex, nucleus, endothelium, and adhesive CTC–EW links (X_C_–X_C_*, X_N_–X_N_*, X_A_–X_A_*, X_E_–X_E_*) are indicated by thick lines. Equations 1–5 define the mathematical frameworks of the Immersed Boundary method.

We use the *IBCell* mathematical framework (the Immersed Boundary model of a Cell; Rejniak, 2007a) to model a two-dimensional (2D) deformable tumor cell traveling through a microvessel. The cell is exposed to both hemodynamic forces exerted by the blood plasma flow, and adhesive–repulsive forces between the tumor cell attaching to or migrating along the EW of the microvessel (**Figure 1**).

### CTC STRUCTURE

The CTC structure is simplified to include two key elements defining cell shape and stiffness: the cell actin cortex and cell nuclear envelope. Both of these intracellular structures are modeled as dense networks of linear Hookean springs indicated by blue links C and gray links N in **Figure 1**, and mathematically defined by Eq. 5. The spring stiffness can be modified either globally (i.e., for each spring forming the nuclear envelope) or locally (i.e., for an individual actin filament in the cortex). This allows us to test their relative role in preserving the overall cell shape under the blood flow as well as in cell attachment and migration capabilities. The whole cell is interpenetrated by the viscous incompressible cytoplasm, but other intracellular elements (such as organelles, microtubules, intermediate filaments) are omitted for simplicity and to reduce computational costs. However, they can be incorporated in this model in a form of additional collections and/or networks of springs.

### EW STRUCTURE

Similarly, the EW is modeled as a mesh of short and relatively stiff linear springs (shown as red links E in **Figure 1** and Eq. 5) that form a uniform rigid wall. For simplicity, no individual endothelial cells are included in the model, but in principle, they can be modeled in a similar fashion as CTCs.

### CTC–EW INTERACTIONS

The CTCs interact with EWs upon contact via the membrane receptors located on both the tumor cell and the endothelium. The proximity between CTC and EW results in the emergence of adhesive links that are modeled as short linear Hookean springs (indicated by green links A in **Figure 1** and Eq. 5). These adhesive links can be dynamically assembled and disassembled based on the distance between CTC’s and EW’s receptors, as well as adhesive spring stiffness. Since the main goal is to investigate cell deformability, we assume that receptor–ligand binding is always effective when the CTC–EW distance is small.

### BLOOD PLASMA FLOW

We do not include the red blood cells or any other non-tumor cells and take into account only the fluid phase of the blood, i.e., the plasma. We model the blood plasma as a viscous incompressible Newtonian fluid governed by the Navier–Stokes equations (**Figure 1**, Eqs 1 and 2). We assume that the velocity of the plasma inside the microvessel has a parabolic profile with zero velocity at the microvessel walls. Thus, in the presence of no other obstacles inside the microvessel, the fluid flow is laminar. However, in the presence of the deformable tumor cells inside the duct, as well as adhesive interactions between the flowing cells and the endothelium, the plasma flow profile may be distorted. For simplicity, we neglect any changes that the plasma flow may have on the endothelium. However, we are aware that the blood flow can, for example, alter the expression of certain membrane receptors in the endothelial cells that in turn may modify the CTC–EW interactions.

### IMMERSED BOUNDARY METHOD

Interactions between the CTCs, EWs, and the plasma flow are solved using the classical fluid–structure interaction approach, i.e., the immersed boundary method (Peskin, 2002 and Eqs 1–5 in **Figure 1**). In this system, Eq. 1 is the Navier–Stokes equation of a viscous incompressible fluid defined on the Cartesian grid ***x*** = (x_1_,x_2_), where *p* is the fluid pressure, μ is the fluid viscosity, ρ is the fluid density, ***u*** is the fluid velocity, and ***f*** is the external force density. Equation 2 is the law of mass balance. Interactions between the fluid and the material points **X**(*l*,*t*) on the tumor cell and the EW boundaries (*l* is an index along either the boundaries of the tumor cells Γ_t_ or the boundaries of the EWs Γ_e_), are defined in Eqs 3 and 4. Here, the force density **F**(*l*,*t*) acting on the cell and wall boundaries is applied to the fluid using the 2D Dirac delta function δ, while all material boundary points **X**(*l*,*t*) are carried along with the fluid. The boundary forces **F**(*l*,*t*) arise from elastic properties of the tumor cell membranes, from rigid properties of the EWs and from CTC–EW adhesion, and are all represented by the short linear Hookean springs in Eq. 5, where *S* is the spring stiffness, *L* is the spring resting length, and **X^⋆^**(*l*,*t*) is the adjacent, opposite, or neighboring point for the elastic, rigid, or adhesive forces, respectively (the pairs of connected boundary points in the endothelium, the EW–CTC adhesion, the CTC nucleus, and cortex are indicated in **Figure 1** by wide red, green, gray, or blue segments, respectively). These equations are solved using the finite difference methods with a discrete approximation of the Dirac delta function and are described in detail in Rejniak (2007a) and Rejniak and Dillon (2007).

## RESULTS

Individual tumor cells in circulation are exposed to the hemodynamic forces and fluid shear that the cells need to withstand in order to keep their physical integrity. The goal of this investigation is to determine the biomechanical properties of the CTCs (either persistent or dynamically changing during the course of CTC voyage through the vascular system) that allow the cells to survive in the blood stream and attach to the EW. First, we examine how the relative stiffness of the CTC cortex versus the stiffness of the CTC nuclear envelope controls CTC deformation under the blood flow (see CTC Survival in the Blood Flow). Next, we test how CTC cytoskeletal properties need to be modified in order to attach and roll on the EW (see CTC Rolling on the Endothelium). Finally, we examine how CTC cytoskeleton reorganization and cell membrane receptor redistribution enable CTC anchorage and migration on the endothelium (see CTC Anchorage to the EW). Since, we are concerned here with the fundamental biophysical parameters of a single deformable tumor cell only, the current model does not include other kinds of cells (such as red blood cells, leukocytes, platelets) that the CTC can collide with, or the formation and dynamics of multicellular emboli. These could be easily added to the model and will be considered in further research.

### CTC SURVIVAL IN THE BLOOD FLOW

**FIGURE 2:**
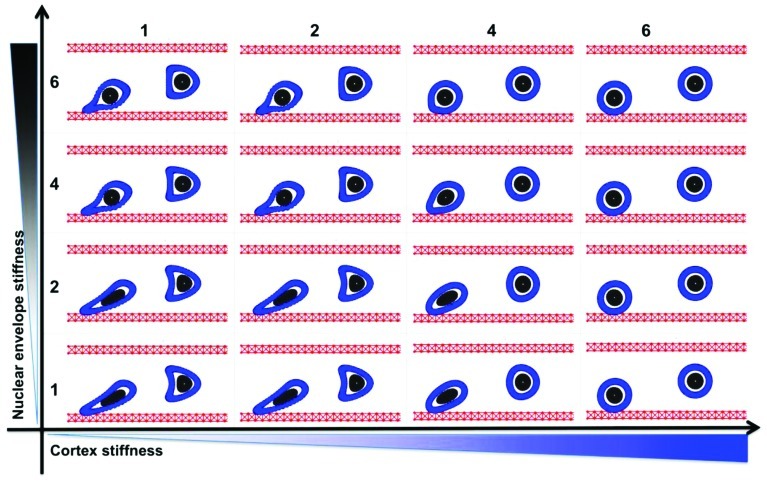
**Circulating tumor cell stiffness relationship.** Circulating tumor cell deformation under steady blood flow in relation to the cell cortex versus cell nuclear envelope stiffness. Final configurations of two separately simulated cells that vary only by their location within the vessel (red) are overexposed on one picture. The cell cortex stiffness (blue) and cell nuclear envelope stiffness (gray) were varied by sixfold each.

We consider a single tumor cell under the blood plasma flow in a small 30-micron-wide and 75-micron-long vessel. The fluid flow is assumed to have a parabolic profile with a maximal velocity of 0.6 mm/s in the center of the microvessel and 0 on the vessel walls. Initially, the cell is circular with a diameter of 10 microns, a 4-micron-wide nucleus, and a 2-micron-wide cortex band. We independently simulated two cases that varied only by initial cell location within the vessel, i.e., whether the cell was placed near the EW or at the middle of the microvessel. The latter cell was exposed to symmetrical hemodynamic forces, whereas the former cell was exposed to non-uniform blood shear stress with increasing blood velocity afar from the EW. We used computer simulations to trace how each cell is carried with the blood flow through the straight segment of the microvessel. Only two model parameters were varied: the stiffness constant of individual fibers forming the cell cortex and the stiffness constant of the cell nuclear envelope. The baseline value in each case was assumed to be 50 dyn/cm and was varied separately over the sixfold range. The final cell shapes after reaching 2/3 of the vessel length was recorded for comparison and overexposed on the same picture. A table showing final cell configurations for the whole range of stiffness constants is shown in **Figure 2**. Except for the case of a very stiff cortex (column 6 in **Figure 2**), when all cells retain their initial circular shapes, the cells located near the EW underwent significant deformations due to the non-uniform blood flow shear stress. However, when the stiffness of the cell cortex is fixed, the increasing stiffness of the cell nuclear envelope results in diminished deformation of the whole cell. This is the most visible in **Figure 2**, column 4, where the difference in the shape of the near-wall cell in the bottom row versus the top row changes from elliptical to circular. A very flexible cortex results in cell elongation that in extreme cases (**Figure 2**, column 1) may resemble cytoplasmic fragmentation (clasmatosis). Thus, actin cortex stiffness is crucial for cell survival during its passive transport by the blood flow, and a combination of soft cortex and soft nuclear envelope may lead to cell damage by the blood stream.

### CTC ROLLING ON THE ENDOTHELIUM

**FIGURE 3:**
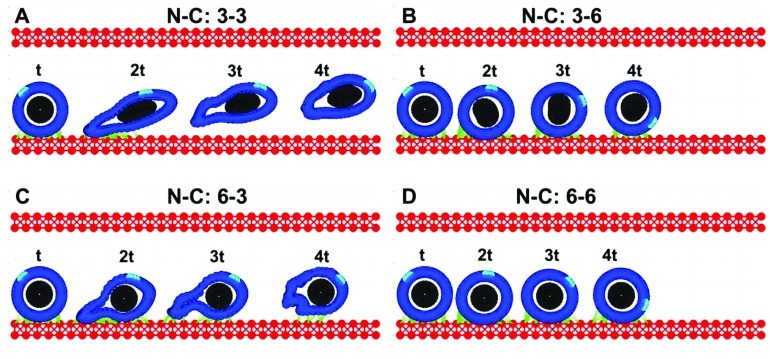
**Circulating tumor cell rolling on the endothelium.** Each of the four cases **(A–D)** is characterized by a different cortex C and nucleus N stiffness value, however the strength of all adhesive bonds is fixed. The values are reported similarly as in **Figure 2**. **(A)** both the cortex and nucleus stiffness are 3-fold higher than the baseline; **(B)** the nucleus stiffness is 3-fold and cortex stiffness is 6-fold higher than the baseline; **(C)** nucleus 6-fold and cortex 3-fold; **(D)** both the nucleus and cortex stiffness is 6-fold higher than the baseline. For each case, four time snapshots (taken at time t, 2t, 3t, and 4t) are overexposed on the same picture for comparison of cell deformation and translocation in each case. A fixed small part of the cell cortex is stained for illustration of the rolling effect.

Based on simulations from the previous section (**Figure 2**), we consider only these combinations of parameters for which the CTCs were able to survive under the blood flow, i.e., the stiffness values for both the cortex and the nucleus from the top-right quarter in **Figure 2**. The goal was to investigate which of these CTCs were able to attach to and roll on the endothelium wall. Four time snapshots from each simulation are shown in **Figures 3A**–**D**. Only in the case of a very stiff cortex (sixfold of the baseline, **Figures 3B**,**D**) the cell was able to roll over the endothelium wall (a fixed part of the cell cortex is stained to show its translocation along the cell perimeter). However, there is a difference in the speed of cell rolling, and a less stiff nucleus contributes to faster cell movement (**Figure 3B**). When both the cell cortex and the nucleus are softer, the cell deforms and is carried with the blood plasma flow (**Figure 3A**). In case of a stiff nucleus and a softer cortex, the cell was initially deformed and attached to the endothelium, but finally, its adhesive connections were broken, and the cell moved apart from the endothelium (**Figure 3C**). These simulations indicate that the transition in the CTC transport phase from floating with the blood flow to attaching and rolling on the EW requires stiffening of the whole CTC cortex. The uniformly flexible cell cytoskeleton prevents the tumor cell from its stable anchoring to the endothelium. In contrast, since the flexible cell becomes more easily elongated due to the blood shear stress, it is more likely that it will be shredded out of the surface in contact and returned to the blood stream. Increased CTC–EW adhesions have been tested but did not improve cell adhesion and rolling abilities (results not shown).

### CTC ANCHORAGE TO THE EW

**FIGURE 4:**
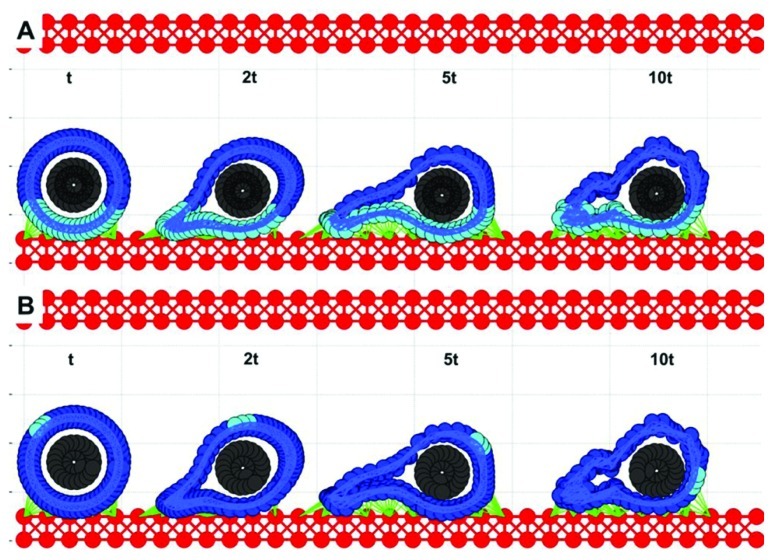
**Circulating tumor cell migrating on the endothelium.** Four time snapshots (taken at time t, 2t, 5t, and 10t) are overexposed on the same picture for comparison of cell deformation and translocation. **(A)** The cell cortex is stained differently depending on its local stiffness: soft (cyan) close to cell focal adhesion with EW, and stiff (blue) far from EW contacts. **(B)** A fixed small part of the cell cortex is stained for illustration of the rolling effect.

Simulations from the previous section (**Figure 3C**) showed that the cell with a relatively stiff nucleus and softer cortex was able to attach to the endothelium and migrate a short distance until it was detached by the flow. We tested the case in which the CTC–EW attachment resulted in further softening of the cell cortex around cell focal adhesions, but was unchanged elsewhere. Four snapshots taken over a 10t period (2.5 times longer than shown in **Figure 3C**) were overexposed on the same picture in **Figures 4A**,**B**. Dynamical changes in the cell cortex allow the cell to migrate successfully on the endothelium when simultaneously exposed to the blood plasma flow. Here, the cortex fibers were softened around the cell focal adhesions with the EW, and they became stiffer immediately after the focal adhesions were broken. All other cortex fibers remained stiff. The softer and stiffer parts of the cell cortex are stained differently in **Figure 4A**. The corresponding images in **Figure 4B** show translocation of a small fixed part of the cell cortex around the cell circumference. These simulations showed that transition from cell rolling, which requires a quite stiff cytoskeleton, to cell anchoring and crawling, in which the contact area between the CTCs and the EWs needs to increase gradually, demands alterations in the cell cortex stiffness. This local softening of the cell cytoskeleton along the contact area with the EW may be potentially modulated by signals from the endothelial cells in contact.

### DYNAMICAL CHANGES IN THE CTC CYTOSKELETON DURING ITS INTRAVENOUS TRANSPORT

**FIGURE 5:**
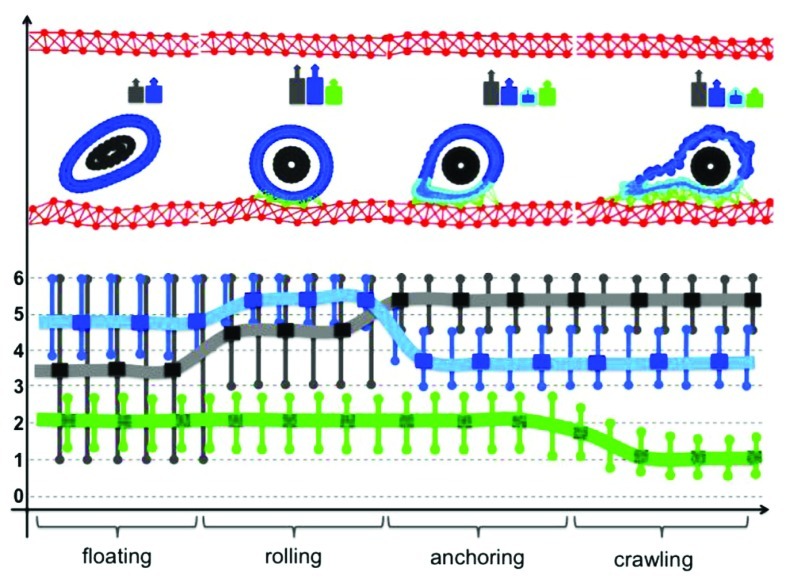
**Dynamical changes in the cell cytoskeleton during CTC intravenous transport.** Each of the steps of the adhesive cascade requires different cytoskeletal properties of the CTC actin cortex stiffness (blue), cortex stiffness near the adhesion sites (cyan), stiffness of the CTC nuclear envelope (gray), and strength of the CTC–EW adhesion bonds (green). A typical cell configuration for each of the steps of the adhesive cascade is shown in the top row together with a color-coded cell stiffness signature. Lines represent the best continuous fit of the computational data.

Simulations of the *IBCell* model revealed that in order for the CTC to progress from floating to rolling, to anchoring, and finally to crawling, the stiffness of its cytoskeleton needs to be modified differently in different cell compartments in a very dynamical way (**Figure 5**). Cell rolling requires quite a stiff actin cortex that can be pushed by the blood flow without extensive deformation. It also demands dynamical assembly–disassembly of CTC–EW adhesion bonds between the cell front and its back. Upon transition to anchoring, the CTC cytoskeleton must become more flexible along the contact surface. Too weak a cytoskeleton leads to cell elongation and its drift with the blood flow; too stiff a cytoskeleton will result in persistent cell rolling. Cell crawling requires weaker adhesive bonds and simultaneous changes in the CTC cytoskeletal stiffness: softening in the cell compartment in contact with the EWs, and stiffening in the cell compartment opposite to the contact surface. These complementary and dynamic changes in the CTC cytoskeletal properties enable the cell to complete the whole adhesive cascade.

## DISCUSSION

Recent experimental studies (Fuhrmann et al., 2011; Swaminathan et al., 2011; Ketene et al., 2012) showed that normal, dysplastic, and metastatic cells (derived either from patient tumors or from cancer cell lines) exhibit a different degree of mechanical stiffness and cell deformability. The overarching conclusion is that even in the earliest stages of cancer development, the mechanical properties of the cells are altered. As cancer cells get progressively more invasive and metastatic, they display softer mechanical characteristics that result in larger cell deformations and more pronounced shape changes. There is also evidence of changes in the structural components of the nuclear envelope in various kinds of cancer cells (reviewed in Chow et al., 2012) that may result in altered mechanical properties of the cells. Numerous *in vivo* studies (Yamauchi et al., 2005; Stoletov et al., 2007, 2010) showed that metastatic tumor cells are quite deformable, and both the cell cytoplasm and cell nucleus can undergo strong compression and shape deformation in small capillaries. In particular, the length of cell nucleus can increase 1.6-fold, and the cell major axis 3.9-fold in comparison to the same cell kind in larger microvessels (Yamauchi et al., 2005). Moreover, the cells arrested at the capillary bifurcation points can stretch and extend their bodies in both directions (**Figure 3**, Yamauchi et al., 2005; **Figure 2**, Stoletov et al., 2010). A change in nuclear morphology is used as a criterion in the current pathological assessment of tumor grade and progression. Nuclear deformations are also observed during tumor cell migration, especially in confined microenvironments, which have been linked to altered rigidity of the cell nuclear envelope and changes in its fiber (laminins, actins, spectrins, titins) composition (Rowat et al., 2008; Friedl et al., 2011; Chow et al., 2012).

However, the precise mechanisms and extent of such deformations in the CTCs in the blood flow are not known. Despite the continuously increasing library of biomechanical assays and sophisticated bioengineering techniques for assessing various properties of cancer cells and their cytoskeleton (reviewed in Suresh, 2007), such precise measurements are difficult to carry in the fluid microenvironment. Most of the currently used methods for capturing CTCs require cell attachment or anchorage to the substrate. This may result in changes in the cytoskeletal properties of the cell in a way that subsequent measurements will not reflect cell properties in circulation. We propose here to use computational simulations to determine cell shape deformations that may be compared to morphologies of CTCs from *in vivo* or *in vitro* studies. This will provide a qualitative not quantitative comparison; nevertheless it may point to possible alterations in cell cytoskeleton stiffness.

The computational *IBCell* framework employed in this paper, has been previously established as a model of a deformable eukaryotic cell (Rejniak, 2007a), and it was used to investigate the formation of abnormal invaginations of the trophoblast tissue in the human placenta (Rejniak et al., 2004), the development of normal and aberrant morphologies of mammary epithelial acini (Rejniak and Anderson, 2008a,b; Rejniak et al., 2010), as well as various emerging patterns of invasive tumor colonies (Rejniak, 2005; Anderson et al., 2009). Here, we used it to model various forms of transport of an individual CTC through the microvessel and CTC–EW interactions. We considered a single cell with a deformable cortex and nucleus and tested parameters related to the actin network and cell nuclear envelope stiffness that allow for successful completion of the adhesion cascade steps. We did not address the kinetics of the receptor–ligand pair, since this mechanism has been extensively simulated by others, but mostly under the assumption that the cells were non-deformable solid spheres. Our goal was to investigate the complementary case. We assumed that cell-endothelium adhesion is effective, but that the cells are deformable. Thus, this model differs significantly from previously published models of cells in circulation. The individual deformable but nucleus-free red blood cells were modeled in (Pozrikidis, 2003, 2005) showing results similar to the soft nucleus cases consider in this paper. The large colonies of interacting red blood cells and platelets under the blood flow were modeled in (Zhang et al., 2009; Crowl and Fogelson, 2011; Zhao et al., 2012), where platelet’s passive translocation within the cellular suspension of red blood cells was investigated. Adhesion and rolling of leukocytes on EWs was modeled in (King and Hammer, 2001; Migliorini et al., 2002; Eniola et al., 2003; Sun et al., 2003; Jadhav et al., 2005; Bose et al., 2010), where various mechanisms of receptor–ligand bond formation and hydrodynamic interactions between individual cells under the blood flow were investigated. However, these models have not addressed all the various modes of intercapillary cell translocation using one comprehensive modeling framework, as has been presented in this paper. Our work shows that the CTC cytoskeleton needs to be dynamically modified in a controlled way to enable the cell to pass through different modes of translocation (i.e., from floating to rolling, to anchoring, to crawling). In particular, the physical properties of the fibers forming the cell actin cortex need to be altered in response to both blood shear stress and adhesive contacts with the endothelium. In the case of cell rolling, we found out that the soft cells were not able to roll in contrast to the stiff cells (such as solid spheres). However, the cell cortex needed to be softened in order for the cell to anchor and crawl on the EW, a case that has not been modeled previously in this context. Such dynamical alterations in cell mechanical properties might be modulated by intercellular signaling and might be a subject of further experimental studies.

**FIGURE 6:**
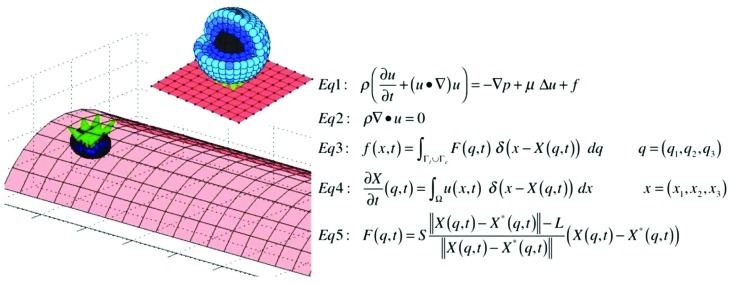
**Three-dimensional model schematics and equations.** A schematic representation of the structure of the 3D cell (left, top), the microvessel with a single attached cell (left, bottom), and model equations in three dimensions (right).

The model described in this paper is 2D, however, this computational framework can be easily extended to 3D (Peskin, 2002; Griffith et al., 2007; Rejniak, 2007b). In the 3D version, the cell cortex and nucleus can be discretized using a dense set of boundary points connected with short linear springs in the form of cross-linked fibers (**Figure 6**). Similar discretization can be used for the boundary of the vessel that together with stiff connecting springs will form a rigid wall (**Figure 6**). The CTC–EW adhesion links can be modeled using short springs like in the 2D case. The immersed boundary equations in both cases have a similar form (**Figures 1** and **6**). One advantage of the 3D model is the possibility of investigating blood vessels of different curvatures and sizes. A disadvantage is in the required computational power. The 3D model needs to be run with a very small time step to ensure the fluid dynamics code stability and convergence, and thus it will take much longer to complete full 3D computations. However, we do not expect that the 2D and 3D models will differ in their qualitative predictions.

Other extensions of the current model can include a comparison of cell deformations under blood flow of different velocities, and in vessels of different size, including small capillaries. We will also test cells of different nucleus-to-cytoplasm ratios, since it has been shown that CTC can vary significantly among each other (Marrinucci et al., 2010). Recent studies indicate that CTCs can form aggregates in the blood system that may also contain other circulating cells, such as leukocytes, macrophages, and platelets (Cho et al., 2012). We will investigate the dynamics of such emboli in the circulation, and whether the presence of other cells can increase the chance of CTC attachment to the EWs. Further experimental studies, out of the scope of our computational group, will be required to determine whether the cell physical properties observed using this computational approach can be manipulated experimentally to disrupt some steps of the adhesive cascade, and may be potentially used to develop the next generation of therapeutic interventions.

## Conflict of Interest Statement

The author declares that the research was conducted in the absence of any commercial or financial relationships that could be construed as a potential conflict of interest.
